# Supplementary material for: Association between the Erythrocyte Membrane Fatty Acid Profile and Cognitive Function in the Overweight and Obese Population Aged from 45 to 75 Years Old
Source: Nutrients. 2022 Feb 21;14(4):914. doi: 10.3390/nu14040914 (PMC8878599; doi:10.3390/nu14040914)
Supplement: Supplementary file 1 [file nutrients-14-00914-s001.zip › nutrients-1589357-supplementary.pdf]

Table S1. The dietary intake fatty acids of the study population in three groups

Variables	NW(n=275)	OW(n=462)	OB(n=337)	*P*
SFAs%	26.196±7.010	26.566±5.760	26.285±5.510	0.476
MUFAs%	35.822±7.620	36.309±5.700	36.258±4.960	0.891
PUFAs%	33.137±8.500	34.513±7.470	34.942±7.010	0.080
UN-Ks%	2.350±1.510	2.286±1.220	2.431±1.090 [b]	0.006**

Data are M ± SD. The discontinuous variable was tested with the Kruskal–Wallis test.. NW: normal weight group; OW: overweight group; OB: obese group; SFAs: saturated fatty acids; MUFAs: monounsaturated fatty acid; PUFAs: polyunsaturated fatty acids; UN-K: unknown; **:*P* <0.01. [b], indicated a significant difference compared with the overweight group.

Table S2. Associations between the scores in all cognitive domains measured by MMSE and the intake of dietary fatty acids in three groups

	Variables	MMSE		MMSE orientation		MMSE memory		MMSE attention		MMSE delayed recall		MMSE language skills	
		B	*P*	B	*P*	B	*P*	B	*P*	B	*P*	B	*P*
**NW**													
	SFAs%	-0.073	0.001**	-0.018	0.035*	-0.013	0.001**	-0.054	0.382	-0.115	0.068	-0.064	0.272
	MUFAs%	0.060	0.331	-0.004	0.946	0.008	0.029*	0.034	0.554	-0.062	0.294	0.007	0.904
	PUFAs%	-0.046	0.403	0.000	0.998	0.065	0.307	-0.045	0.442	0.002	0.967	-0.065	0.254
	UN-Ks%	0.005	0.935	0.043	0.478	0.071	0.238	0.016	0.790	0.063	0.313	-0.044	0.457
**OW**													
	SFAs%	-0.040	0.362	-0.006	0.895	-0.018	0.694	0.005	0.912	-0.019	0.008**	0.015	0.731
	MUFAs%	-0.009	0.844	-0.013	0.785	0.030	0.523	-0.027	0.553	0.035	0.457	0.020	0.639
	PUFAs%	-0.065	0.135	-0.054	0.241	-0.045	0.330	-0.089	0.052	-0.082	0.100	0.002	0.965
	UN-Ks%	0.023	0.595	0.005	0.915	-0.068	0.144	-0.007	0.875	0.028	0.543	0.070	0.043*
**OB**													
	SFAs%	-0.037	0.454	-0.029	0.580	-0.038	0.475	-0.006	0.913	-0.018	0.039*	0.043	0.384
	MUFAs%	-0.032	0.516	-0.006	0.910	-0.069	0.196	-0.039	0.481	0.005	0.929	-0.011	0.826
	PUFAs%	-0.057	0.249	-0.028	0.602	-0.002	0.975	-0.069	0.198	0.000	0.996	-0.050	0.315
	UN-Ks%	-0.004	0.935	0.038	0.472	-0.024	0.646	0.006	0.914	-0.066	0.223	0.014	0.782

Multiple linear regression adjusted for age, gender, BMI, waist-hip ratio, culture, smoking, drinking, exercise, history of hypertension, diabetes mellitus, hypertriglyceridemia, energy intakes. NW: normal weight group; OW: overweight group; OB: obese group; SFAs: saturated fatty acids; MUFAs: monounsaturated fatty acid; PUFAs: polyunsaturated fatty acids; UN-K: unknown; MMSE: Mini-Mental State Examination; *:*P* <0.05; **:*P* <0.01.

Table S3. Associations between the scores in all cognitive domains measured by MoCA and the intake of dietary fatty acids in three groups

Variables	MoCA		MoCA visuospatial function		MoCA naming		MoCA attention		MoCA language skills		MoCA abstracting		MoCA memory		MoCA orientation	
	B	*P*	B	*P*	B	*P*	B	*P*	B	*P*	B	*P*	B	*P*	B	*P*
**NW**																
SFAs%	-0.144	<0.001**	-0.065	0.251	-0.015	0.799	-0.019	0.754	-0.032	<0.001**	-0.022	0.003**	-0.120	0.065	-0.015	0.023*
MUFAs%	0.101	0.003**	-0.117	0.050	0.066	0.271	0.031	0.579	0.035	<0.001**	0.097	0.157	0.024	0.042*	0.018	0.785
PUFAs%	-0.066	0.014*	0.019	0.027*	-0.088	0.138	-0.047	0.404	-0.028	<0.001**	-0.015	0.012*	-0.035	0.544	-0.002	0.974
UN-Ks%	-0.080	0.129	-0.031	0.580	-0.018	0.764	0.010	0.862	-0.089	0.011*	-0.111	0.064	-0.043	0.467	0.032	0.603
**OW**																
SFAs%	-0.035	0.409	-0.027	0.547	0.009	0.848	0.016	0.731	-0.024	0.633	-0.075	0.126	-0.047	0.291	-0.026	0.577
MUFAs%	0.010	0.813	0.016	0.713	-0.017	0.712	0.058	0.193	-0.028	0.553	-0.053	0.251	0.044	0.328	-0.034	0.468
PUFAs%	-0.025	0.548	0.071	0.105	-0.071	0.124	-0.022	0.625	-0.011	0.050	-0.012	0.018*	0.047	0.297	-0.028	0.544
UN-Ks%	0.025	0.548	-0.006	0.892	0.028	0.543	0.031	0.490	-0.012	0.792	-0.035	0.434	0.078	0.082	-0.005	0.922
**OB**																
SFAs%	0.000	0.995	0.036	0.545	0.018	0.742	0.035	0.513	0.052	0.346	-0.111	0.065	-0.028	0.613	-0.013	0.806
MUFAs%	-0.009	0.865	0.067	0.220	0.005	0.925	-0.003	0.955	0.034	0.535	-0.055	0.313	-0.053	0.333	-0.038	0.477
PUFAs%	0.001	0.992	0.023	0.018*	0.075	0.173	0.003	0.954	-0.062	0.256	-0.021	0.001**	0.015	0.778	-0.046	0.397
UN-Ks%	0.031	0.544	0.010	0.854	0.002	0.966	0.051	0.328	0.064	0.243	-0.090	0.091	-0.017	0.754	0.023	0.665

Multiple linear regression adjusted for age, gender, BMI, waist-hip ratio, culture, smoking, drinking, exercise, history of hypertension, diabetes mellitus, hypertriglyceridemia, energy intakes. NW: normal weight group; OW: overweight group; OB: obese group; SFAs: saturated fatty acids; MUFAs: monounsaturated fatty acid; PUFAs: polyunsaturated fatty acids; UN-K: unknown; MoCA: Montreal Cognitive Assessment; *:P <0.05; **:P <0.01.
